# The Interaction Between the Microbiome and Tumors

**DOI:** 10.3389/fcimb.2021.673724

**Published:** 2021-08-31

**Authors:** Yawen Zong, Yujie Zhou, Binyou Liao, Min Liao, Yangyang Shi, Yu Wei, Yuyao Huang, Xuedong Zhou, Lei Cheng, Biao Ren

**Affiliations:** ^1^State Key Laboratory of Oral Diseases & National Clinical Research Center for Oral Diseases, West China School of Stomatology, Sichuan University, Chengdu, China; ^2^Department of Cariology and Endodontics, West China School of Stomatology, Sichuan University, Chengdu, China

**Keywords:** cancer, microbiome, tumorigenesis, tumor progression, gene mutations, metabolism, host immunity

## Abstract

Cancer is a significant global health problem and is characterized by a consistent increase in incidence and mortality rate. Deciphering the etiology and risk factors are essential parts of cancer research. Recently, the altered microbiome has been identified within the tumor microenvironment, tumor tissue, and even nonadjacent environments, which indicates a strong correlation between the microbiome and tumor development. However, the causation and mechanisms of this correlation remain unclear. Herein, we summarized and discussed the interaction between the microbiome and tumor progression. Firstly, the microbiome, which can be located in the tumor microenvironment, inside tumor tissues and in the nonadjacent environment, is different between cancer patients and healthy individuals. Secondly, the tumor can remodel microbial profiles by creating a more beneficial condition for the shifted microbiome. Third, the microbiome can promote tumorigenesis through a direct pathogenic process, including the establishment of an inflammatory environment and its effect on host immunity. The interactions between the microbiome and tumors can promote an understanding of the carcinogenesis and provide novel therapeutic strategies for cancers.

## Introduction

Cancer is one of the most common fatal diseases. In 2018, there were 18.1 million new cases and 9.6 million cancer-related deaths worldwide (Bray et al., 2018). The economic burden from the healthcare of cancer patients was $125 billion per year in the USA and occupied a giant proportion of public health expenditure (Mariotto et al., 2011). Understanding cancer causation and risk factors is an essential part of public health missions. Genetic mutations, infection, tobacco, diet, and radiation, are considered to be common risk factors that promote the development of cancer (Willett, 2000; Anand et al., 2008; Ashford et al., 2015).

Microorganisms are able to colonize the gut, skin, oral cavity (OC), urine, and other environments of human body (Kho and Lal, 2018). The microbiome can be defined as a characteristic microbial community that occupies a reasonable well-defined habitat with distinct physio-chemical properties (Lloyd-Price et al., 2016; Sender et al., 2016; Kho and Lal, 2018; Berg et al., 2020). The microbiome has been developed as a diagnostic marker, a pathogenic agent or a therapeutic target in some diseases due to the rapid development of sequencing technology (Kaczor-Urbanowicz et al., 2017; Gopalakrishnan et al., 2018; Temraz et al., 2019). In cancer, chemotherapy, medications and an altered diet have been shown to significantly influence microbial composition and function (Hayase and Jenq, 2021; Szczyrek et al., 2021). Additionally, the microbiota is highly correlated with type- or subtype-specific tumors (Banerjee et al., 2018; Ma et al., 2019; Nejman et al., 2020). The oncology progression can also remodel the human microbiome (Mima et al., 2015; Burns et al., 2018; Burns and Blekhman, 2019). Recently, Manzoor et al. (2020) proposed a hierarchical relationship between the microbiome and tumors. Specifically, researchers proposed a direct interaction between microorganisms and tumors. Secondly, the microbiome is able to act as a tumor biomarker. Third, the microbiome modulates therapeutic drug efficacy.

In order to better understand the relationship between the microbiome and tumors, we summarized and discussed the relationship between the microbiome and some human cancers, according to their distinct colonization sites. Cancer patients possess a distinct composition of microbiomes located in the tumor microenvironment (TME), inside tumor tissues and in a nonadjacent environment, compared to healthy individuals. Tumor progression can remodel the microbial community, while the human microbiome plays diverse roles in tumorigenesis. The interaction between the microbiome and tumors also highlights novel therapeutic strategies against cancers as the microbiome and some probiotics can affect current cancer treatments in some cases (Khan et al., 2021; Kim and Lim, 2021; Yoon et al., 2021).

## Cancer Patients and Healthy Individuals Have Distinct Microbiomes

In the late 19th century, Robert Koch and Louis Pasteur discovered bacteria inside tumor tissues (Compare and Nardone, 2014). However, the source of where these bacteria emerge remains unclear. In recent years, the microbiome has been proven to be tumor type-specific and plays important roles in tumor development (Banerjee et al., 2018; Ma et al., 2019; Nejman et al., 2020). Currently, the tumor related microbiome research is mainly focused on the microbiota located in the TME, inside the tumor tissue and in the nonadjacent environment.

### Microbiome in the Tumor Microenvironment

TME refers to a complex and dynamic entity containing organs, tissues, their function and metabolism. The TME is highly related to tumor occurrence, growth, and metastasis (Khalaf et al., 2021). The microbiome in TME of colorectal cancer (CRC) has been well-characterized (Rowland, 2009; Ahn et al., 2013; Sears and Garrett, 2014; Cheng et al., 2020). Shah et al. (2018) found an overlap of microbial composition in a tumor biopsy and the paired fecal sample from CRC patients by comparing the microbiome from the tumor biopsies, paired fecal samples, and adjacent tissues. This finding suggests that the fecal microbiome can be an excellent noninvasive biomarker for the CRC. The abundance of some species, including *Peptostreptococcus stomatis* and *Parvimonas micra*, were significantly increased in the feces collected from CRC patients (Zhang et al., 2018b). Zeller et al. (2014) revealed that the CRC-associated fecal microbiota shifted the function from fiber degradation to the utilization of host carbohydrates and amino acids. The CRC-associated imbalance of fecal microbiota also contributed to an enrichment of metabolites (i.e. polyamines) (Yang et al., 2019). The mycobiome refers to the fungal community of the microbiome. (Huffnagle and Noverr, 2013; Seed, 2014; Chin et al., 2020). The mycobiomes were found to be different between the early-stage and late-stage CRCs (Coker et al., 2019). Additionally, the radio from the fungal phylme *Basidiomycota/Ascomycota* was increased among patients with CRC compared to healthy individuals (Gao et al., 2017; Coker et al., 2019; Qin et al., 2021). Except for fungi and bacteria, the virome, composed of endogenous retroviruses, eukaryotic viruses, and bacteriophages (Santiago-Rodriguez and Hollister, 2019), is also related to CRC. Nakatsu et al. (2018) found that *Orthobunyavirus, Tunavirus, Phikzvirus, Betabaculovirus* and *Zindervirus* were represent Eukaryotic viruses in subjects with CRC, while *Fromanvirus* seemed to be represented only in the healthy cohort. Interestingly, there was an significant increase of the diversity of the gut bacteriophage community compared with controls, especially *Streptococcus* phage SpSL1, *Streptococcus* phage 5093, *Streptococcus* phage K13, *Vibrio* phage pYD38-A and *Enterobacteria* phage HK544 (Nakatsu et al., 2018).

The oral microbiome is another main component of the human microbiome and can be collected through a comfortable and noninvasive method (Kaczor-Urbanowicz et al., 2017; Chattopadhyay et al., 2019). Guerrero-Preston et al. (2016) found that head and neck squamous cell carcinomas patients exhibited a significant loss in diversity of microbiota in the saliva. In particular, the family *Enterobacteriaceae* and genus *Oribacterium* can help distinguish oral squamous cell carcinoma (OSCC) samples from oropharyngeal cancer and control samples. In addition to saliva, the periodontal pockets, tooth surfaces and mucosa also harbor various oral microbiomes. The species *Parvimonas micra* and *Neisseria sicca* were associated with a reduced risk of OSCC, while an unnamed *Actinomyces* (oral-taxon_170) was associated with an increased risk (Hayes et al., 2018). Fungi also play important roles in OSCC. Shay et al. (2020) found that *Ascomycota* was the predominant fungus from the oral wash samples of OSCC patients. The abundance of *Candida albicans* and *Rothia mucilaginosa* in OSCC patients were higher compared to healthy individuals, while *Candida dubliniensis, Schizophyllum commune* and a fungus from the class of *Agaricomycetes* were over-represented in healthy controls. *Candida* was proven to be a predominant fungal genus in the oral fungal microflora in some OSCC patients (Mukherjee et al., 2017; Perera et al., 2017; Vesty et al., 2018), indicating a positive relationship between *Candida* and OSCC.

The urogenital tract is also an important microbial habitat (Whiteside et al., 2015). Wu et al. (2018) found a significant difference in the urinary microbial community between the bladder cancer and non-cancerous groups. The microbiome from TME of genital organs is also related to other cancers. Walther-Antonio et al. (2016) discovered that there was a significant difference in the structures of microbiomes from the vagina, cervix, fallopian tubes and ovaries of endometrial cancer. In particular, the species *Atopobium vaginae* and an uncultured *Porphyromonas* sp. were associated with disease status, especially if the vagina had a pH>4.5.

Some tumor engraftment areas are not traditional microbiota-enriched environments. The prostatic fluid lacks prostate cancer-specific microbial species. However, the microbial diversity in patients with high prostate-specific antigen levels is low (Ma et al., 2019), which indicates that the shifted microbiota may break stability of the prostate microenvironment and provide a novel biomarker for patients with high prostate-specific antigen levels.

### Intra-Tumoral Microbiome

Recently, Nejman et al. (2020) performed a comprehensive analysis of the tumor microbiome from 1526 tumor tissues, as well as adjacent normal tissues across several common cancer types, including breast, lung, ovary, pancreas, melanoma, bone, and brain tumors. They demonstrated that tumors contained different bacteria and bacterial contents inside their tumor cells. Notably, the intra-tumoral microbiome represents type- or subtype-specific characteristics, and the highest enrichment appeared in the breast cancer (BC). Previously, Urbaniak et al. (2016) also isolated special bacterial species (*Bacillus, Enterobacteriaceae, Staphylococcus Escherichia coli*, and *Staphylococcus epidermidis*) with a relatively high abundance from BC and identified their DNA-damaging ability in HeLa cells. The malignancy and subtype classifications also represented a strong correlation with the tumor microbiome. Meng et al. (2018) demonstrated that a decrease in the relative abundance of the family *Bacteroidaceae*, while the genus *Agrococcus* increased during BC malignancy. Microbial function predicted by the PICRUS indicated that these bacterial species influenced biotin and glycerophospholipid metabolism, as well as flavonoid biosynthesis. There are four main types of BC, including endocrine receptor (ER)-positive, triple-positive, human epidermal growth factor receptor-2 (Her2)-positive and triple-negative. Banerjee et al. (2018) demonstrated that the triple-positive and triple-negative samples have distinct microbial patterns, while ER-positive and Her2-positive samples share similar microbial signatures by using hierarchical clustering analysis. Microbial diversity is also different between different racial groups. Black women with the higher BC morbidity have an increased abundance of the genus *Ralstonia* in breast tissue compared to white women (Smith et al., 2019). Meanwhile, prostate tissue samples from African men demonstrated an increase of the predominant genera, including *Streptococcus, Alicycliphilus, Acidovorax, Escherichia, Bacteroides, Eubacterium, Parabacteroides, and Odoribacter*, in prostate cancer compared to non-African men (Feng et al., 2019).

Despite a high overlap of microbial abundance among the CRC tumor biopsy and paired fecal samples, approximately 20% of isolated microbiota were different (Shah et al., 2018). For bacterial species inside the CRC tissues, Warren et al. (2013) observed that the dominant bacteria, including the species from the genus *Fusobacterium*, *Leptotrichia*, and *Campylobacter*, were all gram-negative anaerobes, which were previously recognized as common bacteria from the oral microbiome. Although there was no significant microbial difference in either the Topography-Lymph Node-Metastasis stage or clinical tumor stage, Sheng et al. (2020) identified five distinct microbial genera (*Bacteroides, Fusobacterium, Faecalibacterium, Parabacteroides*, and *Ruminococcus 2*) from the proximal and distal CRC segments.

In OSCC, microbial diversity was found to be significantly reduced in tumor tissues, compared to the adjacent normal tissues, saliva, and mouthwash samples (Mukherjee et al., 2017; Zhang Z. et al., 2019). Chng et al. (2016) found that cholangiocarcinoma tumors colonized much more opportunistic pathogens from the genus *Stenotrophomonas*, compared to normal tissues. For other microbes, Carey et al. (2020) discovered that the family double-stranded DNA viruses, specifically *Papillomaviridae*, owned the most viral copies in the primary oropharyngeal squamous cell carcinoma tissues and positive lymph node samples. In most cancer specimens, the viruses, including *Baculoviridae, Reoviridae, Siphoviridae, Myoviridae*, and *Polydnaviridae*, were detected at high levels. Peters et al. (2019) found that lung cancer (LC) tissues had lower microbial richness and diversity, compared to paired normal tissues. There was a negative correlation between microbial diversity of LC-paired normal tissues and cancer survival. In gastric cancer (GC), Yu et al. (2017) found that *Helicobacter pylori* was a dominant species from the microbiota, even in nonmalignant gastric tissue of some GC patients, indicating that *H. pylori* was the primary cause of GC in early stages of neoplastic transformation.

### Microbiome in the Tumor Nonadjacent Environment

The colonization niche of some bacteria has not yet been fully elucidated. It has been proven that the gram-negative bacteria from advanced CRC tumor microbiome are similar to the oral microbiome (Warren et al., 2013). The microbial composition in lungs is also more similar to that of the OC (Yu et al., 2017). Nakatsu et al. (2018) found that the interactions between bacteriophages in fecal samples and oral commensal bacteria from CRC patients performed altered characteristic compared with controls indicating the important roles of virus in CRC. These ectopic microbiomes from the nonadjacent environment have been shown to play important roles in tumor progression.

The whole digestion system provides a natural migration tunnel for microorganisms. There is a significant enrichment of specific intestinal microorganisms (*Bifidobacteriaceae, Enterobacteriaceae*, and *Enterococcaceae* families) in the liver from cholangiocarcinoma tumor tissue (Chng et al., 2016). There was also a significant correlation between the non-digestive cancers (LC, prostate cancer, and multiple myeloma) and the intestinal microbiome (Liss et al., 2018; Liu et al., 2019; Zhang B et al., 2019). Shi et al. (2019) found that the gastrointestinal microbiome was associated with a degree of lymphatic invasion of BC.

Blood circulation is another environment that is influenced by tumor development. The serum microbiome structure of GC patients was significantly different from patients with inflammation compared to healthy controls (Dong et al., 2019). The serum microbiome has the potential to be a biomarker of GC, as it has been shown to have a high correlation with Topography-Lymph Node-Metastasis stage, lymphatic metastasis, tumor diameter and invasion depth (Dong et al., 2019). In biliary tract cancer, the plasma microbiome can be a predictive biomarker due to altered abundance of *Bifidobacteriaceae, Pseudomonaceae* families, *Corynebacterium, Ralstonia*, and *Comamonas* species (Lee et al., 2020).

**Figure 1:**
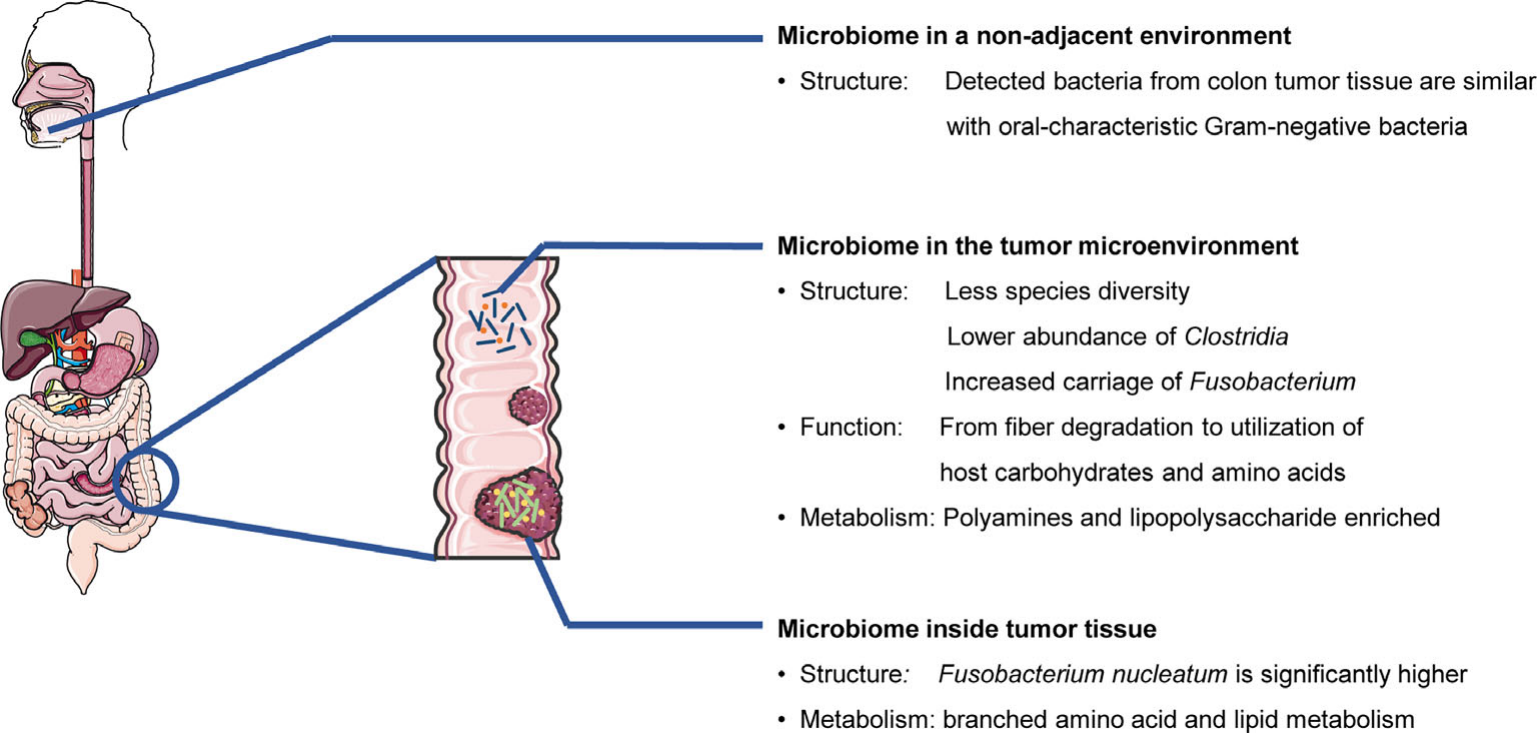
Cancer patients and healthy individuals have different microbiomes. (Colorectal cancer and related bacteria are used as examples.)

In summary, cancer patients at different clinical stages and malignancy tend to have specific characteristics of the microbiomes (structure, function, and metabolism) from the microenvironment, tumor tissues, or nonadjacent microbial locations (**Figure 1**).

## Tumor Progression Affects the Microbial Community

Tumor-specific microbiome highlights whether the tumor progression can reshape the tumor-related microbiome in TME, tumor tissues or the nonadjacent environment (Walther-Antonio et al., 2016; Meng et al., 2018; Shrestha et al., 2018; Zhang et al., 2018b; Dong et al., 2019; Shi et al., 2019). Genetic mutations from tumor cells are considered to be important characteristics for the identification of tumor biomarkers. There are specific genetic mutations across several tumors, including ER and Her2 genes in BC, genes involved in DNA mismatch repair and Sirtuin-3, loss of free fatty acid receptor 2 in CRC, cytokeratin 19 fragment, neuron-specific enolase, carcinoembryonic antigen and Tumor Protein P53 mutations in LC. These mutations were found to be highly correlated with tumor-specific microbiomes (Banerjee et al., 2018; Greathouse et al., 2018; Hale et al., 2018; Zhang et al., 2018a; Liu et al., 2019; Lavoie et al., 2020; Sheng et al., 2020). Burns et al. (2018) validated that loss-of-function mutations, particularly pathway-level mutations, were able to change the predicted interactions in the microbiome. Tumor mutations can have an effect on the whole microbial network, rather than individual microbes (Burns et al., 2018; Burns and Blekhman, 2019).

The TME provides a suitable condition for location of the shifted microbiome. For example, the progression of CRC was found to be accompanied by different ratios of *Fusobacterium nucleatum* (Liesenfeld et al., 2015; Mima et al., 2016). In addition, researchers observed that the CRC tumor resection altered the concentrations of microbial metabolites within urine, and then decreased the abundance of related microbial species. Walther-Antonio et al. (2016) discovered that a high vaginal pH environment was highly correlated with endometrial cancer, as well as the abundance of some species (i.e., *Porphyromonas* sp.). Garza et al. (2020) indicated that the altered microbiota can obtain nutrition from the enriched metabolites within tumor tissue to support their self-growth.

Destruction of the physiological barrier also forms favorable conditions for the microbiome. Yoon et al. (2019) discovered that intestinal uptake was affected by BC and was found to be positively related to the abundance of the *Citrobacter* genus from the *Enterobacteriaceae* family, but negatively related to the unclassified *Ruminococcaceae*. Zhou and Boutros (Zhou and Boutros, 2020) validated that the dysfunction of intestinal barrier, induced by an abnormal activation of c-Jun N-terminal kinase signaling pathway, formed a feedback amplification loop in order to remodel the gut microbiome in a drosophila tumor model.

**Figure 2:**
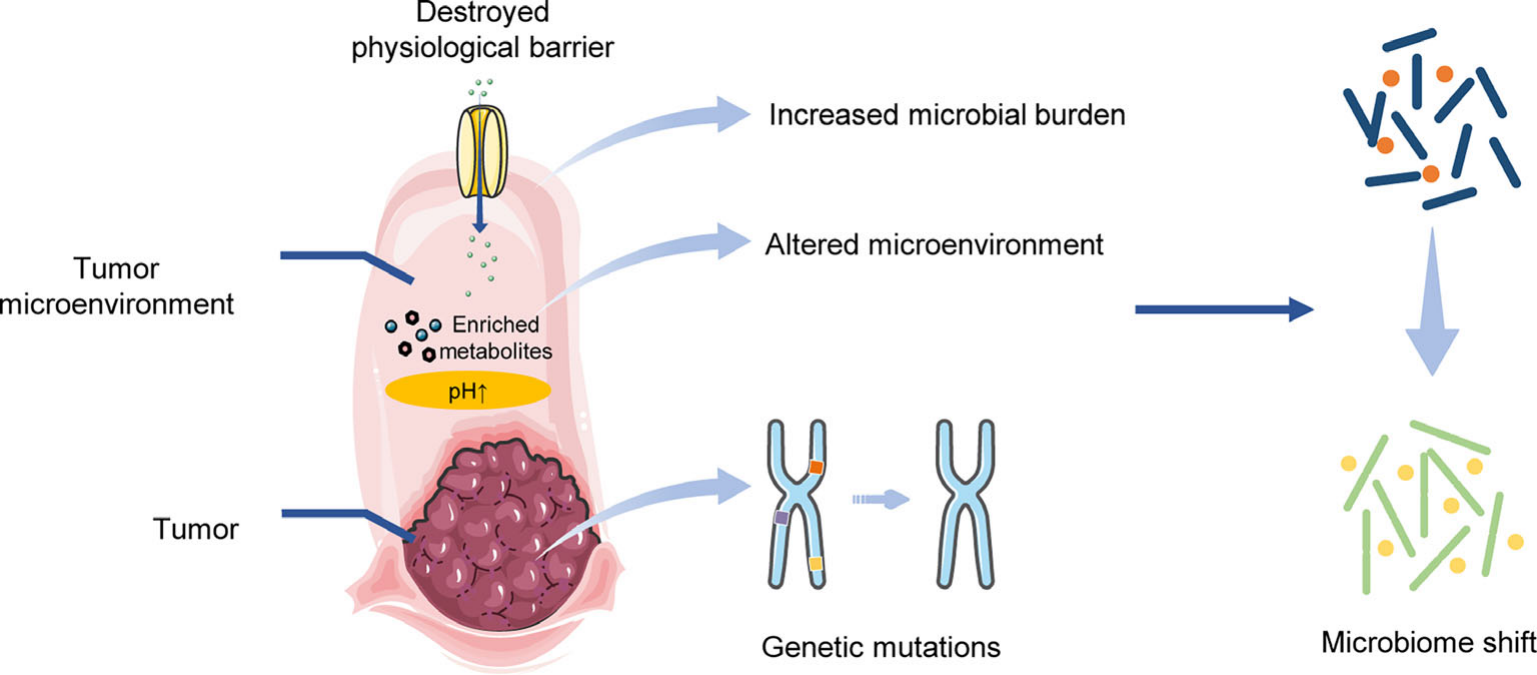
Tumor progression affects the microbial community.

Overall, tumor progression is able to reshape the microbial community. Genetic mutations in tumorigenesis can have an effect on tumor-related microbiome. The tumor microenvironment, including the metabolite enrichment and permeability alterations in the physiological barrier, can provide a niche for the shifted microbiome (**Figure 2**).

## The Microbiome Drives Tumorigenesis

The microbiome has been considered to be a tumorigenic factor since Robert Koch and Louis Pasteur discovered bacteria inside tumor tissues in the late 19th century (Compare and Nardone, 2014). Currently, several microbes have been proven to be important risk factors in tumorigenesis, such as *H. pylori* in GC; *Streptococcus bovis*, *F. nucleatum* and *Porphyromonas gingivalis* in CRC, and *human papilloma* virus in cervical cancer and OSCC (Ellmerich et al., 2000; Humans, I.W.G.o.t.E.o.C.R.t, 2012; Chi et al., 2015; Yamamura et al., 2017; Diaz et al., 2018; de Carvalho et al., 2019; Laniewski et al., 2020; Wang et al., 2021). The tumorigenic effects of the microbiome have been widely investigated over recent years and have suggested that a specific microbial profile (rather than a certain microbe) from the TME, inside tumor tissue or nonadjacent environments can also drive tumorigenesis.

### Microbiome in the Tumor Microenvironment

Several studies have reported that antibiotic treatment can help decrease the number and volume of tumors in mice, including CRC, prostate cancer, pancreatic cancer and melanoma (Zackular et al., 2013; Pushalkar et al., 2018; Sethi et al., 2018; Aykut et al., 2019; Stashenko et al., 2019). The transplantation of the microbiome from tumor-bearing mice or patients into germ-free mice is a practical way of confirming whether the microbiome can drive tumorigenesis. The mice transplanted with a CRC-related microbiome obtained a doubled-tumor burden (Zackular et al., 2013). Baxter et al. (2014) transplanted fecal microbiota from three CRC patients and three healthy individuals into germ-free mice and found that tumor accounts of the mice were strongly related to the microbiota colonized in the mice prior to CRC-induction treatment. Ericsson et al. (2015) validated that different microbiomes can regulate the host burden of CRC. They also observed that there was less butyrate production, but more host glycan degradation from the metabolic pathways of the microbiota in the TME of CRC-susceptible mice, compared to non-susceptible ones. A high concentration of microbial virulence genes was also identified from the CRC intestinal microbiome (Burns et al., 2015). Notably, Stashenko et al. (2019) found the OSCC-related metabolic activities of the oral microbiome were similar, which suggests that the carcinogenic microbial metabolites were non-specific in OSCC.

Metabolic changes within the TME may promote the effects of gene mutations. In a mouse model, several studies have demonstrated that some gene mutations could only cause tumorigenesis within a specific microbial community (Maggio-Price et al., 2006; Seamons et al., 2013; Howe et al., 2018). For example, *Helicobacter* can synergize transforming growth factor-β (TGF-β) deficiency in order to promote the CRC tumorigenesis in mice (Maggio-Price et al., 2006; Daniel et al., 2017). In their models, *Helicobacter* was mainly located within the cecum (Maggio-Price et al., 2006). Functional analysis of the gut microbiome from the CRC TGF-β deficient mice revealed that *Helicobacter* induced an increase in production of lipopolysaccharide (LPS) and oxidative phosphorylation. In addition, the metabolic shift of the gut microbiome from CRC TGF-β deficient mice was highly associated with the host inflammatory response, tumor formation, DNA damage and CRC-related polyamine production (Daniel et al., 2017; Yang et al., 2019).

The microbiome can also induce tumor-related genetic mutations. Hale et al. (2018) found that *Bacteroides fragilis* and the sulfidogenic *Fusobacterium nucleatum* affected the CRC DNA mismatch repair. Zhou and Boutros (Zhou and Boutros, 2020) established an intestinal tumor model in drosophila and identified a c-Jun N-terminal kinase-dependent feedback amplification loop between the tumor and the gut microbiome. Abnormal activation of c-Jun N-terminal kinase signaling induced by the tumor caused dysbiosis of the gut microbiome and dysfunction of the intestinal barrier. Depletion of the microbiome restored intestinal barrier function and reestablished the host-microbiome homeostasis.

In addition, the microbiome can affect host immunity. The combination of TGF-β deficiency and *Helicobacter* infection contributes to an inflammatory environment in the intestine by increasing proliferation of epithelial cells, cyclooxygenase-2-positive CD4+ T cells and macrophages (Maggio-Price et al., 2006). A depletion of the gut microbiome reduced CRC burden in mice, along with increased mature T and B cells (Sethi et al., 2018). The gut microbiome also modulated host immunity by reducing the numbers of interferon gamma-producing (IFN-γ+) T cells and inducing interleukin 17A (IL-17A+) and interleukin 10-producing (IL-10+) T cells to drive tumorigenesis (Sethi et al., 2018). *Helicobacter hepaticus* co-infected with *Hepatitis B virus* recruited innate lymphoid cells and promoted hepatocellular carcinoma (HCC) tumorigenesis through an IFN-γ/p-STAT1 axis (Han et al., 2019). In pancreatic cancer, Pushalkar et al. (2018) found that the pancreatic microbiome induced a reduction of myeloid-derived suppressor cells, an increase in M1 macrophages, and a promotion of TH1 differentiation into CD4(+) T cells and CD8(+) T-cell activation in order to tolerate the host immunity.

### Intra-Tumoral Microbiome

The microbiome inside tumor tissues also plays a role in tumorigenesis. Accumulation of the microbiome inside BC tissues can disturb the proliferation of tumor cells by interfering with hormonal production (Abed et al., 2016). The quorum-sensing molecule from *Pseudomonas aeruginosa*, a pathogen inside the breast, led to a reduction in the survival of BC cells (Allali et al., 2015). The bacterial stress response also depends on cellular malignancy and TME, including oxidative stress. Proal and VanElzakker (Proal and VanElzakker, 2021) explained that bacteria, fungi, and viruses can induce or promote a Warburg-like metabolism in infected host cells in order to meet their own replication and nutritional needs. Nejman et al. (2020) detected tumor type-specific microbiomes in melanoma and breast, lung, ovarian, pancreatic, bone, and brain tumors. Notably, some intra-tumoral bacteria were identified as being intracellular, and were located both in cancer and immune cells, particularly in CD45+ T cells and macrophages. This indicates that the intra-tumoral bacteria can gather immune cells in order to regulate tumor growth. In particular, Gram-positive bacteria were detected only in macrophages, while gram-negative bacteria were rarely detected in cancer cells or in CD45+/CD68− immune cells (Nejman et al., 2020). The distinct locations indicated that immune cells may play microbial type-specific roles in response to intra-tumoral bacteria. However, the detailed mechanisms remain unclear.

Intra-tumoral mycobiome is also correlated to tumorigenesis. Aykut et al. (2019) demonstrated that fungi can be enriched in the tumor environment and are able to induce a carcinogenic effect in the pancreatic ductal adenocarcinoma. Researchers identified a 3000-fold increase in fungal abundance from a tumor compared to normal pancreatic tissue. Their work also demonstrated that pathogenic fungi activated the complement cascade when promoting pancreatic ductal adenocarcinoma.

### Microbiome in the Nonadjacent Environment

The microbiome in a nonadjacent environment can affect tumor development. Yu et al. (2010) validated that there was an accumulation of gut-derived LPS in the circulation of rats with HCC. Knockout of the Toll-like receptor 4 (*TLR4*) gene, a receptor of LPS, limited excessive tumor growth, while the reconstitution of *TLR4* restored hepatic inflammation and tumor cell proliferation. Dapito et al. (2012) found that *TLR4* and intestinal microbiota were required for HCC progression by regulating increased proliferation, expression of the hepatomitogen epiregulin and prevention of cell apoptosis. Deoxycholic acid, a gut metabolite produced by the obesity-induced microbiome, induced senescence-associated secretion in hepatic stellate cells and promoted the development of HCC (Yoshimoto et al., 2013). In BC, Kovacs et al. (2019) noted the oxidative stress induced by lithocholic acid, a metabolite from the gut microbiome, was reduced during oncogenesis, which led to a decrease in the diversity of the intra-tumoral microbiome. These results demonstrated that the nonadjacent microbiome, especially their metabolites, played important roles in establishing an inflammatory or oxidative environment to affect tumorigenesis.

Some microbiomes are not directly related to tumor development, but their metabolites can migrate to a pathological site in order to promote tumorigenesis by causing an altered immune environment through the assistance of immune cells and cytokines. Sethi et al. (2018) depleted the gut microbiota and saw decreased tumor burden in pancreatic cancer and melanoma. However, a lack of mature T and B cells reversed this protection. Moreover, the host was found to have fewer IFN-γ+ T cells, and more IL-17A+ and IL-10+ T cells (Sethi et al., 2018). Ma et al. (2018) reported that bile acid, the important metabolite of the gut microbiota, controlled CXCL16 expression of liver sinusoidal endothelial cells in order to modulate the accumulation of natural killer T cells in HCC, indicating that the intestinal microbiota can implement indirect immunosurveillance in HCC progression.

**Figure 3:**
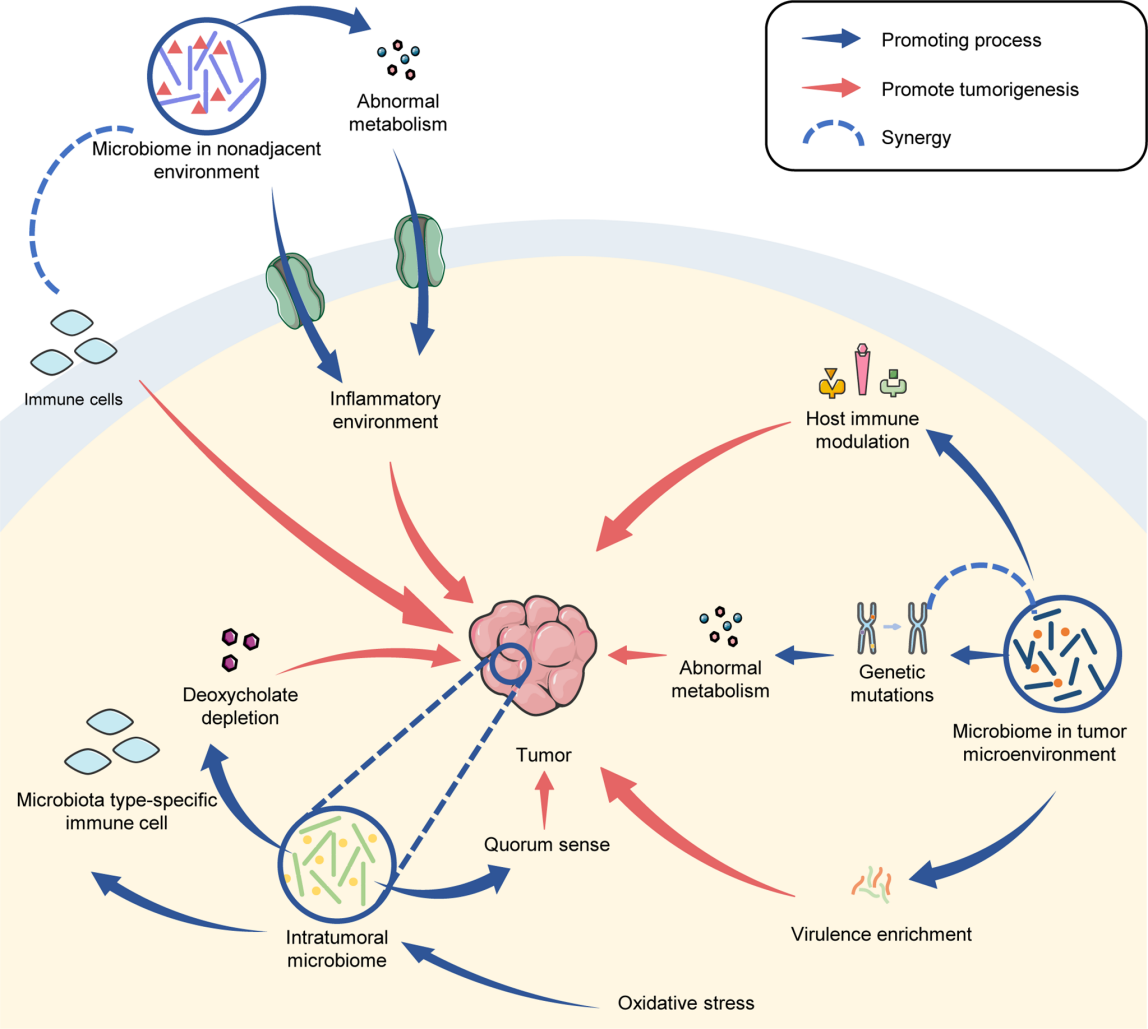
The microbiome drives tumorigenesis.

In conclusion, the microbiome has distinct functions in tumorigenesis that are, to some extent, dependent on its locations. The microbial metabolites and virulence in TME established beneficial conditions for tumor proliferation. The intra-tumoral microbiota affected metabolism, oxidation activity and host immunity to promote tumorigenesis. Furthermore, the microbiota in a nonadjacent environment can induce an inflammatory or oxidative environment through metabolites and affects immune cells in tumor progression (**Figure 3**).

## Discussion

The microbiomes within TME, tumor tissue and even the nonadjacent environment, are specific and play essential roles in tumor development (**Figure 1**). Tumor progression contributes to different architectures of microbial profiles (**Figure 2**), while the shifted microbiome in distinct locations can drive tumorigenesis through direct and indirect effects (**Figure 3**).

However, the interactions between the microbiome and tumor are complicated. For example, despite the microbial type-specific immune cells were identified in several cancers, there was no detailed evidence to prove their function in tumorigenesis (Nejman et al., 2020). The induced signals from the microbiome and their metabolites in a nonadjacent environment have the ability to move to a tumor environment (Yu et al., 2010; Dapito et al., 2012; Yoshimoto et al., 2013; Kovacs et al., 2019). However, the detailed mechanisms are still unclear, including how the translocation occurs, how the translocation affects the microbiome and virulence, and how the translocation affects the carcinogenic process.

The microbiome has been considered to be a risk factor for tumorigenesis since bacteria were discovered inside tumor tissues (Compare and Nardone, 2014). One of the important challenges for the investigation of a tumor-related microbiome is microbial contamination. Several studies described different structures of the microbiome in the proximal and distant sites of tumor, which were considered as the microbiome from tumor microenvironment (Ahn et al., 2013; Mima et al., 2016). Nejman et al. (2020) constructed lots of controls to reduce contamination. Recently, the Cancer Microbiome Atlas provided a protocol to control for sample contamination (Dohlman et al., 2021). It is critical to set up rigorous contamination controls, and choose the proper sample collection sites, as well as types of microbial analysis, to investigate the tumor microbiome.

Currently, the tumor-related microbiota has been mainly focused on bacteria. Other microbes also play essential roles in tumorigenesis, including fungi, viruses, and bacteriophages. The mycobiome has distinct characteristics in cancer patients and healthy individuals. Additionally, it is known to play important roles in tumor development (Gao et al., 2017; Mukherjee et al., 2017; Perera et al., 2017; Vesty et al., 2018; Coker et al., 2019; Qin et al., 2021). Furthermore, the interaction between the mycobiome and microbiome has a critical function in tumorigenesis (Lambooij et al., 2017; Sanchez-Alonzo et al., 2020; Santus et al., 2021). Zapatka et al. (2020) systemically utilized whole-genome and whole-transcriptome sequencing data from 2,658 cancers across 38 tumor types and validated a high prevalence of known tumor-associated viruses, including *Epstein–Barr* virus, *hepatitis* B virus and *human papillomavirus*. These results revealed that impaired antiviral defense may drive tumorigenesis. Recently, Thaker et al. (2019) discussed the impact of virus on tumor metabolism, and showed that the virus, such as *Adenovirus*, *Herpes* family, and *Flaviviruses*, caused different metabolic nodes to remodel the metabolism. Meanwhile, the phages might reshape the microbiome to affect the cancer progression. Hannigan et al. (2018) evaluated the differences of the virome and bacterial community compositions in human CRC. They found that CRC-related bacteriophage communities potentially impacted the tumorigenesis by shifting the bacterial community. Nakatsu et al. (2018) also found the related shift of the interaction between the bacteriophage and oral bacteria. This kind of shifted interactions seemed also appeared in the non-adjacent community.

The efficacy of current cancer therapies, including chemotherapy, radiotherapy and surgery, are highly correlated to the microbial phenotype (Muls et al., 2017; Paul et al., 2017; Nakatsuji et al., 2018; Wang et al., 2018; Lauka et al., 2019; McGee et al., 2019; Wu et al., 2019; Xu et al., 2020a). The microbiome can also influence effectiveness of the immunotherapy (Matson et al., 2018; Uribe-Herranz et al., 2018; Strouse et al., 2019; Wojas-Krawczyk et al., 2019; Xu et al., 2020b). Metabolites of the microbiome also affect treatment outcomes (Hatae et al., 2020; Nomura et al., 2020). Therefore, the microbiome can serve as a potential biomarker or target to distinguish the precision therapeutic strategies for different cancer patients (Hargadon, 2017; Shaikh et al., 2019; Liss et al., 2020; Liu et al., 2020; Song et al., 2020). Meanwhile, the human commensal bacteria, such as *Lactobacilli* and *Bifidobacteria* have also been suggested to play important roles in preventing and treating various tumor malignancies, indicating that the microbiome can be a source of potential therapeutics, as well as a therapeutic target (Motevaseli et al., 2017; Wei et al., 2018; Legesse Bedada et al., 2020; Zuo et al., 2020).

## Author Contributions

XZ, LC, and BR conceptualized the study. YWZ, YJZ, BL, ML, YS, YW, and YH prepared the original draft. XZ, LC, and BR wrote, reviewed, and edited. All authors contributed to the article and approved the submitted version.

## Funding

This work was supported by National Natural Science Foundation of China grant, grant number 81870778 (BR), 81600858 (BR), 82071106 (LC); Applied Basic Research Programs of Sichuan Province (2020YJ0227).

## Conflict of Interest

The authors declare that the research was conducted in the absence of any commercial or financial relationships that could be construed as a potential conflict of interest.

## Publisher’s Note

All claims expressed in this article are solely those of the authors and do not necessarily represent those of their affiliated organizations, or those of the publisher, the editors and the reviewers. Any product that may be evaluated in this article, or claim that may be made by its manufacturer, is not guaranteed or endorsed by the publisher.
